# Platelet-Rich Fibrin-Conditioned Medium as an Alternative to Fetal Bovine Serum Promotes Osteogenesis of Human Dental Pulp Stem Cells

**DOI:** 10.3390/bioengineering10101196

**Published:** 2023-10-14

**Authors:** Ayano Hatori, Daiki Yamakawa, Sarah Al-Maawi, Eva Dohle, Jin Chikira, Yasuyuki Fujii, Megumu Miki, Robert Sader, Daichi Chikazu, Shahram Ghanaati, Yoko Kawase-Koga

**Affiliations:** 1 Department of Oral and Maxillofacial Surgery, Tokyo Medical University, 6-7-1 Nishishinjuku, Shinjuku-ku, Tokyo 160-0023, Japan; ayano.h.1o19@gmail.com (A.H.); daiki19@tokyo-med.ac.jp (D.Y.); jin_c@tokyo-med.ac.jp (J.C.); yasuyuki.fujii0730@gmail.com (Y.F.); 050403megu@gmail.com (M.M.); chikazu@tokyo-med.ac.jp (D.C.); 2 FORM, Frankfurt Oral Regenerative Medicine, Clinic for Maxillofacial and Plastic Surgery, Goethe University, Theodor-Stern-Kai 7, 60596 Frankfurt, Germanyeva.dohle@kgu.de (E.D.); sader@em.uni-frankfurt.de (R.S.); shahram.ghanaati@kgu.de (S.G.); 3 Division of Maxillofacial Surgery and Stomatology, Department of Oral and Maxillofacial Surgery, School of Medicine, Tokyo Women’s Medical University, 8-1 Kawadachou, Shinjuku-ku, Tokyo 162-8666, Japan

**Keywords:** platelet-rich fibrin, human dental pulp stem cells, osteogenic differentiation, mesenchymal stem cells, xeno-free medium, tissue engineering

## Abstract

Human dental pulp stem cells (DPSCs) exhibit multilineage differentiation capabilities and superior clonogenic and proliferative properties. However, the use of animal-derived components such as FBS raises concerns regarding the clinical application of stem-cell-based therapies. Platelet-rich fibrin (PRF) derived from human blood is rich in fibrin, platelets, and growth factors and acts as a bioactive scaffold for grafting with biomaterials. In this study, we assessed the efficacy of PRF-conditioned medium (CM) in promoting DPSCs proliferation and osteogenic differentiation compared with the standard culture medium supplemented with FBS. A comparison of DPSCs cultured in FBS and PRF-CM revealed no differences in characteristics or morphology. However, cells cultured with PRF-CM exhibited inferior proliferation rates and cell numbers during passage in comparison with those cultured with FBS. In contrast, DPSCs cultured in PRF-CM showed significantly higher levels of calcification, and RT-PCR confirmed that the gene expression levels of markers associated with osteoblast differentiation were significantly increased. The PRF-CM approach offers a convenient, straightforward, and advantageous method for culturing DPSCs, without relying on animal-derived components. In summary, this study introduces a novel application of PRF-CM for enhancing the osteogenesis of DPSCs, which provides an alternative to FBS culture medium and addresses concerns associated with the use of animal-derived components in clinical settings.

## 1. Introduction

Dental pulp stem cells (DPSCs) and mesenchymal stem cells (MSCs) of human origin possess the ability to differentiate into various types of cells, such as osteoblasts, odontoblasts, adipocytes, chondrocytes, and cells with neuron-like characteristics [1,2,3]. In comparison with bone marrow stem cells (BMSCs), DPSCs have displayed a higher capacity for clonogenicity and proliferation [1]. Furthermore, DPSCs can be readily procured from extracted teeth using minimally invasive procedures, bypassing ethical concerns [4]. Therefore, the application of DPSCs in cell-based therapies holds significant value for diverse regenerative purposes, especially in the context of bone tissue regeneration, as several studies have shown their elevated propensity for osteogenic differentiation [5]. In our prior research, we confirmed that the use of a DPSC sheet without a scaffold facilitates bone regeneration in a model of bone defect, while simultaneously encouraging bone formation at fracture locations in a mouse model [6,7,8]. Furthermore, another study showcased the efficacy of dense collagen gel scaffolds loaded with DPSCs in enhancing bone healing in a rat model with a critical-size bone defect [9]. In general, the conventional culture method for DPSCs involves using high concentrations (10–20%) of serum, such as fetal bovine serum (FBS), to maintain cell viability. However, the clinical implementation of stem-cell-based therapy requires careful consideration of animal-derived components that pose a potential risk of transmitting pathogens and triggering immune responses in recipients. FBS contains not only growth factors, nutrients, and hormones but also an array of uncharacterized constituents with varying compositions, which can transfer pathogens that disrupt standardized cell preparations [10]. We previously established a novel method for the isolation and proliferation of DPSCs, along with their differentiation into neurospheres under xeno-/serum-free conditions during primary culture using supplements and growth factors [11]. In contrast, we found that the self-renewal capacity of DPSC-derived neurospheres was constrained in the absence of FBS [11]. Therefore, there is a need to develop a new culture method using xeno-free media for DPSCs. Platelet-rich fibrin (PRF) is derived from human peripheral blood and constitutes a system of concentrated blood components obtained through centrifugation without the addition of anticoagulants or external chemicals [12]. PRF can be combined with biomaterials, such as membranes, scaffolds, or bone substitutes to facilitate the infusion of acellular biomaterials with autologous cells and growth factors, thereby enhancing and accelerating regenerative procedures within the implantation bed [13]. PRF has been reported to be successful in surgical cases, such as sinus lift procedures, extraction socket preservation, addressing periapical abscesses, and bone augmentation in accordance with guided bone regeneration (GBR) [14]. In comparison with platelet-rich plasma (PRP), PRF is a more economical, straightforward, and practically applicable option in daily clinical practice [15]. PRF is composed of a densely packed fibrin complex consisting of leukocytes, cytokines, and glycoproteins, such as thrombospondin [16]. We also found that the incorporation of PRF enhanced the regenerative potential of PCL-Mesh in vitro [17]. Moreover, in our prior investigation, we examined how injectable PRF influences the capacity for osteogenic differentiation in a coculture system comprising primary osteoblasts (pOB) and endothelial cells. We demonstrated that injectable PRF (i-PRF) enables the uniform dispersion of platelets throughout the fibrin scaffold, consequently amplifying the number of enclosed platelets and leukocytes [12]. Another study showed that, in comparison with PRP preparations, both advanced PRF (A-PRF) and concentrated growth factor (CGF) extracts contained comparable or higher levels of platelets and platelet-derived growth factors [18].

In recent years, many researchers have adjusted their culture settings to include human platelet lysate (hPL), which stimulates MSC proliferation and differentiation. hPL is produced by disrupting the platelet membrane from human platelet components through repetitive cycles of freezing and thawing. However, the definition of such culture supplements is ambiguous, and batch-to-batch variations in hPL can result from varying plasma quantities, a diverse spectrum of growth factors, and donor-specific influences [19]. To prevent the gelatinization of hPL medium, anticoagulants such as heparin must be added because of the plasma components of hPL, and their concentration must be standardized [20].

Accordingly, we hypothesized that the use of PRF instead of FBS or hPL would promote DPSC growth and osteogenic differentiation. The aim of this study was to evaluate the effectiveness of PRF-conditioned medium (PRF-CM) in comparison with FBS general medium in promoting DPSC proliferation and differentiation capacity.

## 2. Materials and Methods

### 2.1. The Isolation of Liquid-PRF and Preparation of PRF-Conditioned Medium

**Figure 1:**
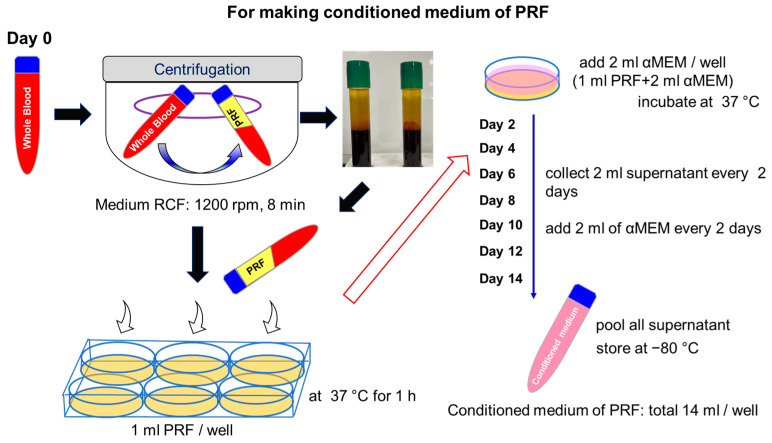
Workflow to make the conditioned medium of PRF. The PRF clot is isolated by centrifugation from blood samples that are subsequently processed at a medium speed of 1200 rpm (177× *g*) for 8 min. Liquid-PRF of 1 mL clots are transferred to 6-well plates at 37 °C for 1 h. Then, 2 mL of αMEM is gently added, and 2 mL of supernatant is collected every 2 days. Instead of supernatant, 2 mL of αMEM was used. This photo shows the actual postcentrifugal S-PRF tubes.

Blood samples were obtained from three individuals (male and female) who were in good health of between 29 and 47 years of age. These donors provided written informed consent to participate in this study. The protocol and consent process of the present study were approved by the Institutional Review Board of the School of Medicine, Tokyo Women’s Medical University Medicine, Tokyo, Japan on 22 July 2022 (Approval No, 2022-029) and Tokyo Medical University, Tokyo, Japan on 11 November 2022 (Approval no. E2022-0324). Blood samples were drawn by venipuncture and collected in glass-coated plastic tubes (S-PRF Tubes, A-PRF, Process for PRF, Nice, France). The samples were immediately centrifuged (Duo centrifuge, Process for PRF, Nice, France) at a medium speed of 1200 rpm (177× *g*) for 8 min. Liquid-PRF (L-PRF) was removed from the tubes using pipettes and separated from the red blood cell phase. The L-PRF was transferred to 6-well plates (1 mL per well) and incubated at 37 °C for 1 h. Next, 2 mL of αMEM was gently added to each well, and the plates were stored at 37 °C for 2 days. After 2 days, the supernatants were collected (2 mL per well) and stored at −80 °C. The αMEM was then replaced with fresh medium every two days until 14 days of PRF cultivation (Figure 1). All collected supernatants were pooled and used as PRF-conditioned medium (PRF-CM). PRF-CM was added at the same concentration as a substitute for FBS in subsequent experiments.

### 2.2. The Isolation and Culturing of Dental Pulp Stem Cells

The present study was conducted after obtaining written consent from all patients and approval from the relevant institutional review boards (as noted above). DPSCs were sourced from the dental pulp of wisdom teeth extracted from four patients (male, n = 2; female, n = 2) of 26 to 31 years of age at Tokyo Medical University Hospital. The dental pulp was initially extracted and finely fragmented, followed by digestion in a solution containing 3 mg/mL collagenase type I (Sigma-Aldrich, St. Louis, MO, USA) for 45 min at 37 °C. Then, single-cell suspensions were obtained by passing the cells through a 70 µm cell strainer. These cells were subsequently seeded onto 100 mm dishes (seeding density: of 1 × 10^5^ cells) and maintained in alpha minimum essential medium (αMEM; Gibco/BRL, Cheshire, UK) supplemented with 1% penicillin–streptomycin–amphotericin B suspension (PSA; Wako Pure Chemical Industries, Osaka, Japan), 15% fetal bovine serum (FBS; Biowest, Nuaillé, France), or 15% PRF-CM. Passage of the cells was carried out when they reached 70% confluence. All experimental procedures utilized DPSCs between passages 3 and 5.

### 2.3. Cell Proliferation

Dental pulp stem cells were cultured at a density of 1 × 10^5^ cells until they reached 70% confluence in 100 mm dishes and passaged with each culture medium (αMEM, 15% FBS, 15% PRF-CM, 1% PSA). For the cell proliferation experiments, cells were seeded at a density of 1 × 10^4^ cells per 96-well plate. The proliferation of DPSCs was assessed by collecting and counting cells at passages 1, 2, and 3 in each culture medium. The proliferation rate was assessed using a Cell Counting Kit-8 (Dojindo, Kumamoto, Japan). The labeling mixture was introduced. After incubation for 3 h at 37 °C, under 5% CO₂, the cells were counted using an EnSpire Multimode Plate Reader (PerkinElmer, Waltham, MA, USA) at 450 nm at 24 h, 48 h, 72 h, 5 days, 7 days, and 14 days of culture.

### 2.4. Osteogenic Differentiation

The cells were cultured in 12-well plates containing an osteogenic differentiation medium. This medium was composed of α-MEM supplemented with either 10% FBS or 10% PRF-CM, 1% PSA, 10 nM dexamethasone (Dex; from Wako Pure Chemical Industries, Osaka, Japan), 10 mM β-glycerophosphate (β-GP; from Sigma-Aldrich, St. Louis, MO, USA), and 100 µM L-ascorbate-2-phosphate (AsAp; from Wako Pure Chemical Industries, Osaka, Japan). This approach was based on a previously published method [21]. The osteogenic differentiation medium was replaced every 3 days, and the cells were maintained for 14 days.

### 2.5. Flow Cytometry

To obtain single-cell suspensions, cells isolated from cultured DPSCs in each of the culture media were suspended in 0.25% trypsin, washed with PBS, then filtered through a 70 µm cell strainer. After this, the cells were blocked using 10% FBS in PBS for 30 min at 37 °C, then incubated at 4 °C for 90 min with fluorescein in phycoerythrin (PE)-conjugated human CD14, CD29, CD34, CD44, CD81, CD90, and CD105 antibodies (BioLegend, San Diego, CA, USA). The control group consisted of cells that were not exposed to the fluorescent antibodies. The cells were then washed and fixed in 4% paraformaldehyde (PFA) for 10 min at 4 °C. A subsequent analysis was performed using a FACS Verse flow cytometer (BD Biosciences, San Jose, CA, USA) in conjunction with the FACS software program (ver. 8.1, FlowJo 10; BD Becton Dickinson, Franklin Lakes, NJ, USA).

### 2.6. Alkaline Phosphatase Staining

Following a 14-day culture in an osteogenic differentiation medium, DPSCs were subjected to alkaline phosphatase (ALP) staining. Briefly, cells were washed with PBS, fixed using 70% ethanol, and then stained for 10 min with a solution that consisted of 0.01% naphthol AS-MX phosphate (Sigma-Aldrich, St. Louis, MO, USA) using 1% N,N-dimethyl formamide (Wako Pure Chemical Industries, Osaka, Japan) as a substrate and 0.06% Fast BB salt (Sigma-Aldrich, St. Louis, MO, USA) as a coupler. Quantification of the relative staining intensity was performed using the ImageJ software program (ver. 1.53, National Institutes of Health, Bethesda, MD, USA), with calculations performed using the Prism 9 software program (ver. 3.1, GraphPad Software, San Diego, CA, USA).

### 2.7. Von Kossa Staining

Following a 14-day culture in an osteogenic differentiation medium, DPSCs underwent a series of steps. First, they were washed with PBS and then fixed with 100% ethanol for 10 min. Subsequently, the cells were rinsed with distilled water and immersed in a 5% silver nitrate solution (Wako Pure Chemical Industries, Osaka, Japan). Afterward, the cells were exposed to UV light for 30 min and then rinsed with distilled water. Finally, a 5% thiosulfate solution (Wako Pure Chemical Industries, Osaka, Japan) was applied for 3 min, followed by another rinse with water. The ImageJ software program was used for the quantification of relative staining intensity, with calculations performed using the Prism 9 software program.

### 2.8. Alizarin Red S Staining

DPSCs were subjected to a 14-day culture in an osteogenic differentiation medium. Following this, they were washed with Ca^2+^-free PBS, then fixed in 10% formaldehyde solution in PBS for 10 min at temperature of 4 °C. After rinsing with distilled water, the cells were incubated in Alizarin Red S solution (Wako Pure Chemical Industries, Osaka, Japan) for 15 min at room temperature to detect calcification. The ImageJ software program was used for the quantification of relative staining intensity, with calculations performed using the Prism 9 software program.

### 2.9. Real-Time Reverse Transcription–Polymerase Chain Reaction (RT-PCR) Analysis

To analyze gene expression, total RNA was isolated from DPSCs that were cultured in differentiation medium using ISOGEN (Invitrogen, Carlsbad, MA, USA). Reverse transcription was performed with a QuantiTect Reverse Transcription kit (Qiagen, Venlo, The Netherlands) in accordance with the manufacturer’s instructions. Real-time reverse transcription polymerase chain reaction (RT-PCR) was performed on a Light Cycler 96 (Roche Diagnostics, Basel, Switzerland) using THUNDERBIRD Next SYBR qPCR Mix (TOYOBO, Osaka, Japan). The RT-PCR conditions were as follows: 95 °C for 60 s, then 45 cycles of 95 °C for 10 s, 65 °C for 30 s, and 72 °C for 45 s. GAPDH served as the internal control, and each reaction was replicated three times. Differences in the relative expression among samples were calculated by the ΔΔCT method. All primer sequences are listed in Table 1.

### 2.10. Statistical Analysis

The Prism 9 software program was used to perform all statistical analyses. The standard deviation of the mean (SD) is indicated by an error bar. Statistical analyses between two groups were performed using a paired Student’s *t*-test. *p*-values of <0.05 were considered to indicate statistical significance (* *p* < 0.05, ** *p* < 0.01, *** *p* < 0.001). All experiments were performed in triplicate.

## 3. Results

### 3.1. Characterization of DPSCs in FBS or PRF-CM Conditions

**Figure 2:**
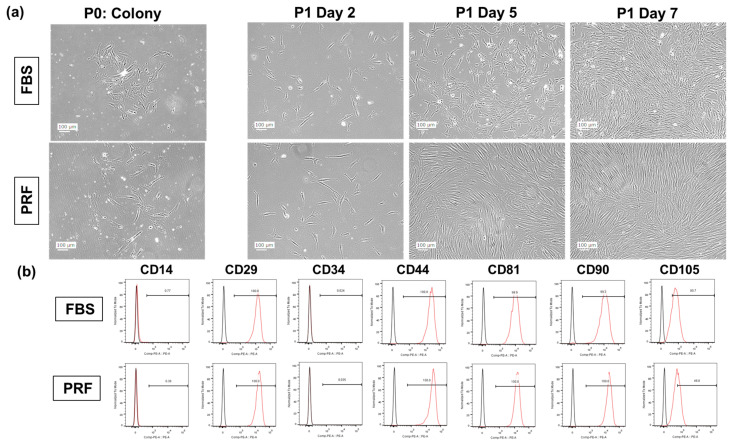
Characterization of FBS and PRF-CM groups in DPSCs: (**a**) Both groups of DPSC shapes were compared at P0 and P1. Cells from the same patient were cultured separately in FBS and PRF. These images were enhanced in Photoshop to improve the resolution. (**b**) Flow cytometry was conducted to detect CD14, CD29, CD34, CD44, CD81, CD90, and CD105 in order to compare the characteristics of the DPSCs.

First, we compared cell morphology and colony formation speed during primary culture in response to FBS- and PRF-CM-supplemented culture medium. Both groups of treated DPSCs exhibited similar spindle-shaped morphologies and growth rates across passages 0–3 (Figure 2a). To further characterize the DPSCs under both conditions, we performed flow cytometry to analyze the expression of cell surface molecules. The analysis revealed that the expression patterns of mesenchymal stem cell surface markers for the DPSCs cultivated in the two media were similar. The DPSCs were negative for CD14 and CD34 and positive for CD29, CD44, CD81, CD90, and CD105 (Figure 2b). 

### 3.2. Comparison of the Proliferation Potential of DPSCs in FBS and PRF-CM

**Figure 3:**
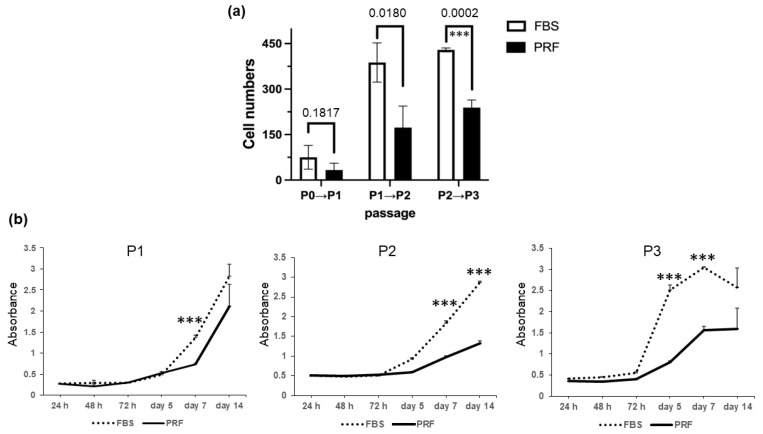
Comparison of proliferative potential of DPSCs in FBS and PRF-CM groups: (**a**) Cell counts of DPSCs were compared between P0 and P2. (**b**) Proliferation speeds were compared between P1 and P3 at time points of 24 h, 48 h, 72 h, 5 days, 7 days, and 14 days. Error bars represent the SD. Statistical analyses were conducted using a paired *t*-test (*** *p* < 0.0001).

The number of DPSCs at passage 0 (P0) in the two experimental groups did not differ to a statistically significant extent. However, in P1 and P2, there were differences in the number of cells for each medium, the number of cells that were cultured in FBS was significantly higher than the number of cells that were cultured in PRF-CM (Figure 3a). Moreover, a colorimetric assay was utilized to investigate the proliferation capacity at 24 h, 48 h, 72 h, 5 days, 7 days, and 14 days of culture in P1 to P3 (Figure 3b). As shown in Figure 3b, there were significant differences in proliferation rates from P1 to P3 depending on the medium supplements used for cultivation, specifically from the time points of days 5, 7, and 14. These results indicate that DPSCs cultured in PRF-CM exhibit lower cell numbers and proliferation rates than DPSCs cultured in FBS.

### 3.3. PRF-CM Promotes Osteogenic Differentiation of DPSCs Compared with FBS

**Figure 4:**
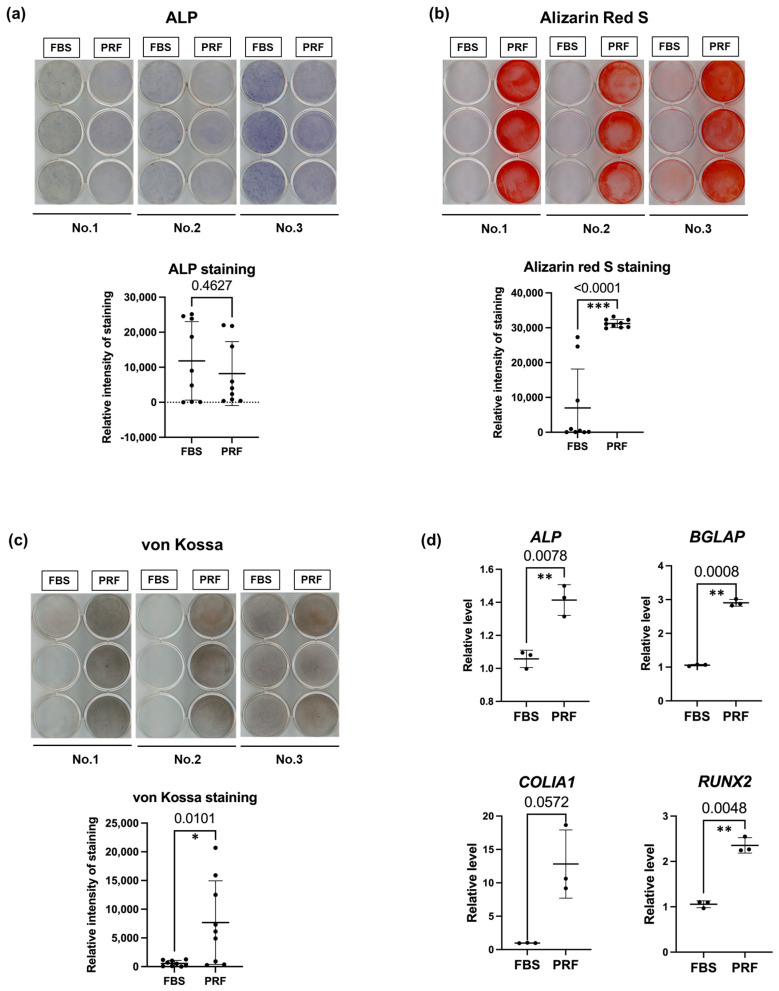
Comparison of the osteogenic differentiation ability of DPSCs in the FBS and PRF-CM groups: (**a**) ALP staining of DPSCs after 14-day culture. Quantification of ALP staining of FBS and PRF-CM groups using ImageJ. (**b**) Alizarin Red S staining of DPSCs after 14-day culture. Quantification of Alizarin Red S staining of both groups using ImageJ. (**c**) Von Kossa staining of DPSCs cultured for 14 days. Quantification of von Kossa staining in the two groups using the ImageJ software program. (**d**) RT-PCR results of the osteogenic markers of both groups in DPSCs cultured in an osteogenic medium with FBS or PRF-CM. Error bars indicate the SD. Statistical comparisons were performed using an unpaired *t*-test (* *p* < 0.05, ** *p* < 0.01, *** *p* < 0.0001).

The osteogenic differentiation potential of the two groups of treated DPSCs was analyzed. ALP, von Kossa, and Alizarin Red S staining were performed after 14 days of cultivation. ALP staining on day 14 revealed slight differences in ALP activity between the two groups (Figure 4a). In comparison, Alizarin Red S staining and von Kossa staining in cells cultured in PRF-CM showed clearly higher calcification than in the FBS groups (Figure 4b,c). Quantification by the ImageJ software program indicated a significant difference between the FBS- and PRF-CM-treated groups in both ALP and von Kossa staining (Figure 4b,c). RT-PCR revealed significantly higher gene expression levels of ALP, osteocalcin (BGLAP), type I collagen alpha 1 (COLIA1), and RUNX2 in the PRF group (Figure 4d).

## 4. Discussion

Dental pulp stem cells (DPSCs) have garnered attention as promising candidates for cell therapy in bone defect regeneration. A recent meta-analysis suggested that the combination of DPSCs or SHED and a scaffold promoted significantly more bone regeneration than a cell-free scaffold, irrespective of the scaffold type and animal species used [22]. The establishment of a xeno-free culture method for DPSCs can expand the range of applications of regenerative cell therapy using DPSCs. In the present study, we compared the efficacy of platelet-rich fibrin (PRF) by preparing PRF-conditioned medium (PRF-CM) obtained through medium-speed relative centrifugation (RCF) and adding it to the culture medium as an alternative to fetal bovine serum (FBS). These results show that there was no difference in the timing or ability to passage between using PRF-CM from primary culture and traditional methods using FBS, although the growth rate and cell number were slightly lower in PRF-CM. However, DPSCs cultured in PRF-CM exhibited enhanced differentiation induction in comparison with those cultured in FBS, suggesting that PRF-CM may promote the osteogenic differentiation of DPSCs. To our knowledge, this is a novel finding, demonstrating that PRF-CM is sufficiently effective to conduct experiments entirely without the use of FBS, starting from the isolation and primary culture stages. Furthermore, in terms of osteogenic differentiation, PRF-CM has proven to be even more effective than gold-standard culture with FBS.

Previous studies comparing serum-free medium with FBS have used various growth factors and recombinant proteins as supplements [23]. In contrast, in the present study, only the same percentages of PRF-CM for growth and differentiation media as FBS were required, making it a simple and safe culture method. Furthermore, a previous study using liquid-PRF in osteo-/odontoblastic induction medium demonstrated that PRF-treated groups exhibited elevated expression of Ki-67 and PCNA, increased ALP activity, and a stronger induction effect at higher doses. This study also indicated that PRF facilitated the osteo-/odontoblastic differentiation of DPSCs, as evidenced by the increased protein and mRNA levels of osteo-/odontoblastic markers [24]. In the present study, Alizarin Red S staining, von Kossa staining, and the RT-PCR results of *ALP*, *BGLAP*, *COLIA1*, and *RUNX2* in cells cultured in PRF-CM show significantly higher calcification than in the FBS group (Figure 4b,c). However, the results of ALP staining show no significant increase in the PRF-CM group compared with the FBS group (Figure 4a). We believe this finding is reasonable, as ALP is a marker of early osteogenic differentiation and is also expressed by preosteoblast progenitor cells. These findings indicate that PRF-CM notably enhances the initiation of osteogenic differentiation, consistent with earlier research, underscoring the potential of PRF-CM for regulating the osteoblast differentiation pathway in DPSCs and suggesting a promising therapeutic approach for promoting bone regeneration and repair.

Moreover, we conducted experiments on neurogenic and adipogenic differentiation using PRF, although the data were not included in this study. For neurogenic differentiation, growth was not satisfactory with PRF-CM, largely due to the low serum content in the differentiation medium, resulting in unclear outcomes. Furthermore, a previous study reported that preconditioning DPSCs with PRF-derived factors did not enhance their neuroregenerative effect [25]. One possible explanation is that undifferentiated DPSCs inherently exhibit several of the molecular markers used for identifying mature neurons, such as Tuj1 [26], which may make it difficult to evaluate the effect of PRF-CM on neuronal differentiation using immunocytochemistry. For adipogenic differentiation, it was difficult to quantify fat droplets after 21 days of adipogenic differentiation. A previous study reported that DPSCs have inferior adipogenic capacity in comparison with dental follicle stem cells (DFSCs) and periodontal ligament stem cells (PLSCs) [27]. Further investigations and experiments are necessary for a more comprehensive and detailed assessment of the multidifferentiation potential of both DPSCs groups.

This study was associated with some limitations. First, it remains unclear why the proliferative and differentiation potential of DPSCs differs when cultured in FBS and PRF-CM. It is possible that the lower proliferation potential observed in comparison with FBS is because PRF-CM is added to the culture medium at the same ratio as pure FBS, resulting in higher dilution in the PRF group (i.e., 15%). This is because 15% of the RPF obtained from blood was not directly added to the culture medium; however, it was prepared as a conditioned medium. In this context, a previous study evaluated the influence of PRF-CM (centrifuged at 44× *g* for 8 min) without any further dilution in cell culture medium (i.e., 100%) in comparison with FBS [28]. The results show a significantly higher cell number in the PRF-CM group in comparison with those treated with FBS. Similar results were observed for cell viability. However, it is also important to note that the DPSCs treated with PRF-CM in our study did not show any negative effects on cell survival or differentiation, leading to the conclusion that PRF-CM used at this concentration (15%) may be rather beneficial for supporting cell differentiation.

Similarly, further studies have investigated the effect of PRF on the differentiation and function of cells when treated with PRF in different fields. A recent study showed that primary osteoblasts cocultured with outgrowth endothelial cells exhibited significantly higher functionality in building vascular-like networks when combined with PRF in comparison with standard cell culture medium [12].

Further research involving larger sample sizes and additional generations may provide more insights into the mechanisms underlying the effects of PRF-CM on DPSCs and potentially lead to applications in regenerative medicine.

Moreover, we previously reported that with a reduction in centrifugation speed at 600 rpm (44× *g*) for 8 min, the release of growth factors from leukocytes and platelets residing within the solid PRF matrices could be enhanced [29,30]. However, considering that a slow rotation rate reduces the amount of liquid-PRF collected, it is important to carefully consider the culture conditions when using low PRF-CM for cell culture. Future studies could also explore the optimal culture conditions for low PRF-CM and compare the effects of different RCF speeds of PRF on DPSCs.

Applying the results of this research could potentially revolutionize bone-regenerative therapies. By obtaining a blood sample from donors beforehand and subsequently harvesting DPSCs from the same donors to cultivate with PRF-CM, it may pave the way for a reality where self-contained cell transplants are possible, eliminating the need to use components derived from different individuals. This development could lead to more coherent and synergistic treatment approaches.

Further research in this area could offer valuable knowledge about the intricate molecular mechanisms underlying the effects of PRF-CM on DPSCs and their potential applications in regenerative medicine.

## 5. Conclusions

In this study, we investigated the use of PRF as a cell culture supplement instead of FBS and evaluated the effect of PRF-CM on the proliferation and differentiation ability of DPSCs. The PRF-CM method for culturing DPSCs is a convenient, simple, and highly useful method that does not involve animal-derived components. Overall, this study contributes to our understanding of the potential of PRF-CM in enhancing the differentiation capacity of DPSCs and its potential implications in regenerative medicine.

## Figures and Tables

**Table 1:** Primer sequences utilized for quantitative real-time PCR.

Gene	Primer Sequences (Forward and Reverse, 5′-3′)	Accession
*GAPDH*	GAAGGTGAAGGTCGGAGTCA	BC023632
	GAAGATGGTGATGGGATTTC	
*ALP*	ATGAAGGAAAAGCCAAGCAG	NM_000478
	ATGGAGACATTCTCTCGTTC	
*RUNX2*	CAGACCAGCAGCACTCCATA	NM_004348
	CAGCGTCAACACCATCATTC	
*COLIA1*	GTGCTAAAGGTGCCAATGGT	NM_000088
	CTCCTCGCTTTCCTTCCTCT	
*BGLAP*	GGCAGCGAGGTAGTGAAGAG	NM_199173
	AGCAGAGCGACACCCTAGAC	

## Data Availability

All data presented in this research can be obtained upon a reasonable request directed to the corresponding author.
